# Neuraxial Anesthesia and Risk of Root Damage: A 3D Ex Vivo Study

**DOI:** 10.3390/neurosci5040044

**Published:** 2024-12-03

**Authors:** Hipólito Labandeyra, Xavier Sala-Blanch, Alberto Prats-Galino, Anna Puigdellívol-Sánchez

**Affiliations:** 1 Laboratory of Surgical Neuroanatomy (LSNA), Human Anatomy and Embryology Unit, Faculty of Medicine and Health Sciences, Universitat de Barcelona, 08036 Barcelona, Spain; hipolitolabandeyra@gmail.com (H.L.); xavi.sala.blanch@gmail.com (X.S.-B.); aprats@ub.edu (A.P.-G.); 2 Division of Regional Anesthesia, Anesthesiology Service, HM Delfos Hospital, 08023 Barcelona, Spain; 3 Division of Regional Anesthesia, Anesthesiology Service, Hospital Clínic de Barcelona, 08036 Barcelona, Spain; 4 Medicina de Família, CAP Anton de Borja-Centre Universitari, Consorci Sanitari de Terrassa, 08191 Rubí, Spain

**Keywords:** regional anesthesia, needle insertion, angles, 3D reconstruction, ex vivo

## Abstract

Cauda equina nerve roots may become damaged during neuraxial anesthesia, and post-puncture headache may appear in the case of cerebrospinal fluid leakage if needle tips are deformed due to bone contact when several attempts are needed. Our aim was to verify the correlation between skin–transverse process distance (st) and skin–dural sac distance (d) for calculation of optimal angles in a free visual guide and as a reference for the maximal depth to be traversed by the needle. Randomly selected ex vivo samples (*n* = 10) were flexed to reproduce the position of the lumbosacral spine during spinal anesthesia. Spinal needles were inserted perpendicular to the skin either blindly or following the inferred paramedian angle corresponding to ultrasound-measured (d). After computed tomography and three-dimensional reconstruction, both (st) and (d) were measured, and the Pearson correlation index was calculated. A free 3D-PDF tool was used to illustrate the potential affectation of nerve cuffs by needles located lateral to the dural sac. Correlation between (d) and (st) was 0.84–0.93 at L4L5-L3L4 intervertebral levels, and most needle tips were located within the spinal canal, but some traversed the zone where nerve cuffs emerge. In conclusion, ultrasound may determine if a perpendicular needle insertion is viable at midline. If not, the optimal paramedian angle and maximal depth may be determined by measuring (st).

## 1. Introduction

The subarachnoid block is the most widely used technique for regional anesthesia in elderly patients [1,2], but it is also widely used in Caesarean anesthesia [3]. Its complications may be reduced by the use of ultrasound [1,4,5,6]. However, the ultrasound visualization of only the posterior complex of dura mater is a predictor of difficult spinal anesthesia [4].

Cauda equina nerve roots may become damaged during neuraxial anesthesia [7,8]. Furthermore, when multiple puncture attempts are required [9], needle tips may become deformed due to bone contact, altering the final shape of the dural sac hole and increasing the likelihood of post-puncture headache if cerebrospinal fluid leakage occurs [10,11]—a common complication also observed in labor anesthesia [3,12].

Needle access to the spinal canal has classically been described in autopsy specimens lying in the left lateral position either from 90° to 100° in the sagittal plane or from 120° to 135° for paramedial insertion [13].

The lateral decubitus flexed fetal position has also been adopted in a magnetic resonance imaging study with a volunteer (height, 158 cm) to better simulate clinical spinal correction and augmentation of the interspinous process distance [14]. That case revealed that median approaches were consistently viable when directed perpendicular to the lumbar skin, below the upper spinous process of each intervertebral lumbar level. Optimal angles for 1–2 cm paramedial approaches, leading to the midline of the dorsal dural sac, were also measured. The maximal and minimal paramedian angles for successful puncture differed by less than 5° from the optimal angles. This led to the development of a formula that depended on the skin-to-dural sac distance (d) and Pythagorean principles. 

Clinical experience shows a first-attempt success rate of only 52.9% [15], with approximately one-third of patients requiring up to five needle redirections and one-fifth needing more than ten attempts or multiple needle insertions to access the intrathecal space. In contrast, pre-procedural ultrasound-guided paramedian approaches that use Pythagorean formulas to estimate insertion angles significantly reduce the number of needle insertion attempts [16]. These findings, along with the progressive reduction in interlaminar space that occurs with age [17], which limits the median approach, suggest a need for greater precision and individualization in punctures in paramedian approaches.

**Figure 1:**
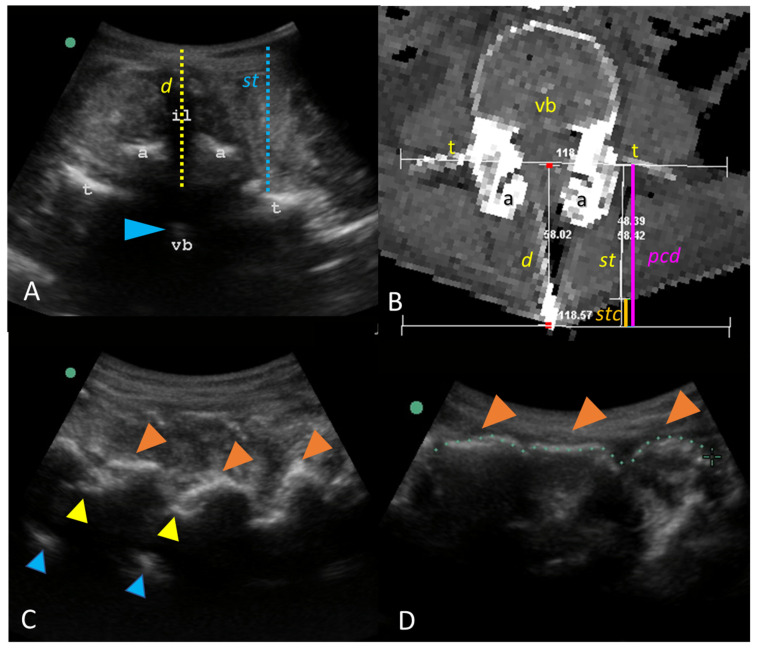
Ultrasound and CT measurements. (**A**) Axial view of the interspinous space. A slight hyperdense shadow corresponding to the vertebral body (vb)—arrowhead- is suggestive of the deepest level of the vertebral canal, delimitated between transverse (t) and articular (a) processes. The skin-to-dural sac distance (d)—discontinuous yellow line- may be determined across the dark shadow corresponding to the interspinous ligament (il). Note that it is comparable to the distance between the skin and the transverse process (st)—blue discontinuous line-. (**B**) Corresponding specular CT image of the same structures in an oblique view, perpendicular to the skin. The reduced (st) in thin patients was corrected by adding (stc)—orange segment- to obtain a parallel corrected distance (pcd)—magenta segment- comparable to the distance (d). (**C**) Best parasagittal oblique view. The deepest hyperechoic image (blue arrowhead) is suggestive of the level of the (vb) when the ultrasound can traverse the flavum ligament (yellow arrowhead) but not the spinous process (orange arrowhead). (**D**) Midline of the same case shows adjacent spinous processes, with a reduced interspinous space (common in older people) that precludes perpendicular needle insertion.

We have developed a three-dimensional (3D) interactive model to illustrate the structures of interest in spinal anesthesia [18,19] and have prepared an experimental free visual guide (CC BY), available at the Public Repository of the University of Barcelona, that may be downloaded in (http://diposit.ub.edu/dspace/handle/2445/179594, (accessed on 29 November 2024)) [20], based on Pythagorean principles. The guide allows the visualization of the estimated optimal angles depending on the (d) measurements [21] (Figure 1).

Since the interspinous ligament may be calcified, measuring (d) by ultrasound may be challenging, as calcification interferes with ultrasound transmission within the tissue. Alternative anatomical measurements, such as the skin-to-transverse process distance, will be explored here to determine the correlation between (d) and the skin-to-transverse process distance (st) when direct estimation of the skin-to-dural sac distance at the midline is not possible. Furthermore, we aimed to verify the needle location in the spinal canal after perpendicular median needle insertion and after applying the visual guide for experimental paramedian approaches in flexed ex vivo trunks, that reproduce the lumbosacral spine position during spinal anesthesia.

## 2. Methods

### 2.1. Study Design

In accordance with the Declaration of Helsinki, the descriptive study of anatomical anesthetic approaches was approved by the ethical committee of the University of Barcelona (IRB00003099). Patient consent was waived because we used unidentifiable samples and images of randomly selected ex vivo specimens from the Donor Service of the Anatomy and Embryology Unit of the Faculty of Medicine. 

The trunks were examined the day after their arrival at the Donor Service. They were stored at 4 °C until tests confirmed the absence of transmissible viruses. No specific preservation processes were applied to maintain maximum similarity to skin resistance during needle insertion, mimicking in vivo conditions. After needle insertion, the trunks were frozen until the moment of the Computed Tomography (CT) scan.

### 2.2. Flexed Lumbosacral Position 

The trunks of the specimens (*n* = 10) were placed in a flexed prone position, using a bearer below the abdomen, to reproduce the lumbosacral spine position during spinal anesthesia.

The skin-to-dural sac distance (d) (Figure 1A) in the interspinous space was determined by ultrasound. The interspinous space is frequently identified as a black shadow, corresponding to the spinous ligament (il), in the absence of a spinous process shadow (Figure 1B,C, orange arrowheads). The distance from the skin to the transverse process (st) of the inferior vertebra of a given interspace was also quantified (Figure 1A).

### 2.3. Visual Guidance of Needle Insertion

**Figure 2:**
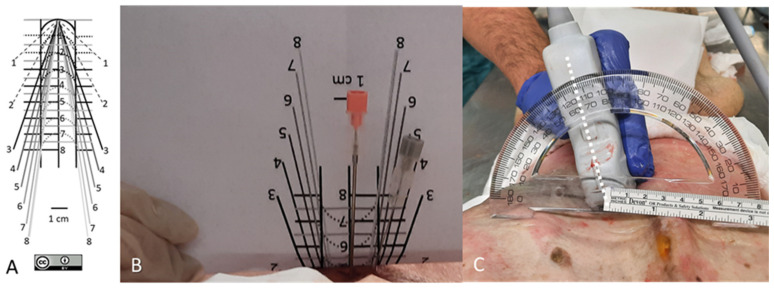
Representative images showing patient positioning and insertion guide. (**A**) Free visual guide for 1 cm paramedian approaches [20]. (**B**) The guide is folded based on the horizontal line corresponding to the skin–dural sac distance (d), as measured by ultrasound (5 cm in this specific case), and the needle is inserted 1 cm paramedially by following the oblique line corresponding to that distance. (**C**) The paramedian distance and the needle insertion angle following the axis of the ultrasound transducer (discontinuous line) may be individualized by searching for the best view of the interlaminar window.

After folding the visual guide (Figure 2A) along the horizontal line corresponding to (d), spinal needles were inserted at 0° relative to the axial plane. The insertion followed the oblique line at a 1 cm paramedian angle corresponding to (d), or alternatively, the median approach was used (Figure 2B), both without continuous ultrasound guidance. In other cases, the paramedian distance was individualized by optimizing the view of the interlaminar window. The needle was then inserted along the ultrasound-determined axis once the probe was removed. (Figure 2C).

### 2.4. Needle Insertion

Needles were inserted perpendicular to the skin, below the upper spinous process, in the median and paramedian approaches. If a bone hit was perceived, the needle was redirected slightly. After a maximum of three slight redirections, the needle could be completely redirected at the anesthesiologist’s discretion, which could include common cephalad angles related to the lower process and variable paramedian distances.

**Figure 3:**
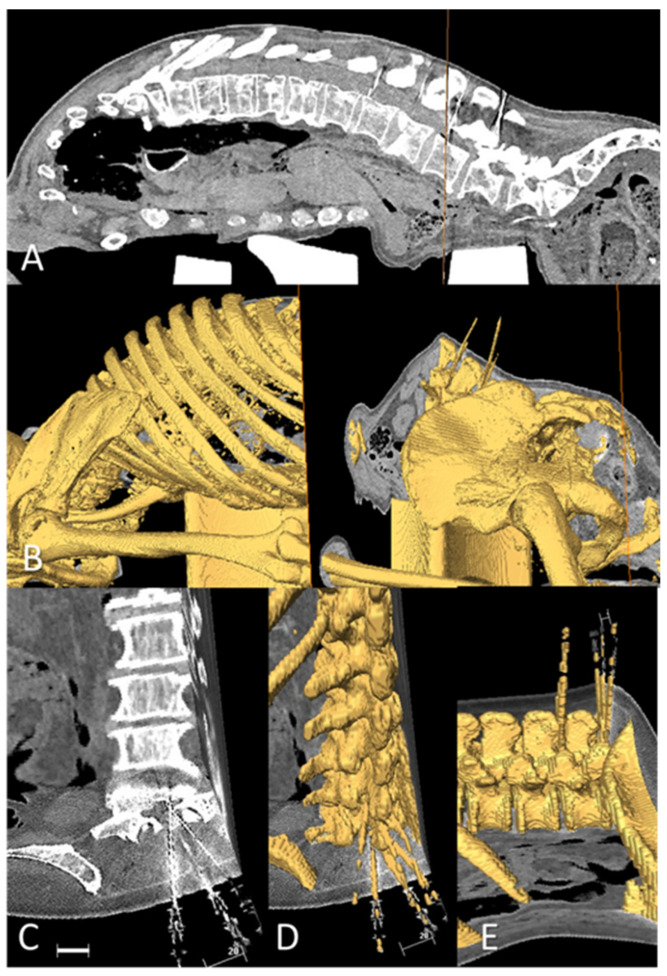
CT and 3D reconstruction. (**A**) Sagittal CT scan showing the flexed trunk and some perpendicular needles inserted in several interspinous spaces. The axial plane of the TC (vertical orange line at the level of the L3 vertebral body) may not coincide with the path of the needle, inserted perpendicular below the upper spinous process of each intervertebral level. (**B**) A 3D reconstruction of the ex vivo sample with a bearer below the abdomen to reproduce the flexed posture. Sagittal and axial illustrative planes are also shown. (**C**) CT slices were examined in three planes to check needle location. Oblique views adapted to the needle paths are prepared to visualize the traversed structures. The paths traversed by the metallic needles are easily visualized as white on X-ray (scale bar 20 mm). Needles are visibly located within the spinal canal when inserted 1 cm paramedially (right side) or at an individualized paramedian distance of 2 cm (left side). (**D**) The 3D software allowed reconstruction by threshold selection, with the bone or needles being denser than surrounding structures. (**E**) Needles may be inserted at 0°, following the axial plane below the upper spinous process when the interspinous space is opened in a flexed trunk with preserved vertebral bodies.

**Figure 4:**
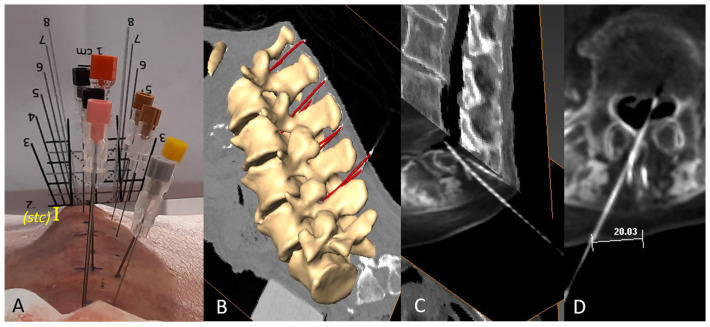
Pilot case: Representative images of successful needle insertions. (**A**) Multiple perpendicular needle insertions were performed in the pilot case, with the guide folded at the skin-to-dural sac distance (d). Needles were inserted following the oblique line corresponding to (d) at the 1 cm paramedian level, within a theoretical plane aligned with the top of the spinous process, even if the final insertion point was closer to the midline. For measurements of st (distance to the transverse process), the same guide can be used to infer the distance from the top of the spinous process to the needle insertion point—here, an stc of approximately 0.5 cm—which is then added to the st measurements to calculate the insertion angle. (**B**) A 3D reconstruction of vertebral volumes of interest and of the successful perpendicular needle insertions. (**C**) Angulation of a paramedially inserted needle within the tissue, finally reaching the dural sac. When the plane of needle insertion does not coincide with the axial TC plane, oblique views adapted to the needle insertion plane were prepared for a better identification of the location of the tip of the needle. (**D**) Correct placement of the needle in the central part of the dorsal dural sac, inserted at an individualized paramedial angle and distance.

Needles were left in the injected zone to assess their final location by computed tomography (CT) (Figure 3). In two cases, the spinous processes were located by palpation and the needle was introduced blindly below the rostral spinous process and perpendicular to the skin. In another two pilot cases, 22–29-gage (G) spinal needles were inserted at T12–L1 to increase the number of guided needle insertions (Figure 4A) after estimating (d). Otherwise, 25 G needles were inserted at the L4–L5 to L2–L3 intervertebral spaces (the most used needles in our service). In another two cases, the optimal paramedial interlaminar window was determined, and the needle was inserted along the ultrasound-determined axis.

Overall, we attempted 22 median, 36 paramedian (32 at 1 cm and 4 free), and 4 individualized paramedian needle insertions, and 2 trunks were used for measurements without needle insertion.

### 2.5. CT and 3D Reconstruction

Conventional CT was performed of the full lumbosacral area in the flexed position (Figure 3A). CT scans were performed using the Somatom Sensation 64 system (Siemens Medical System, Erlangen, Germany). Digital Imaging and Communications in Medicine (DICOM) files were analyzed using 3D editor software designed for visualizing and analyzing biomedical images (Amira 5.3, Mercury Co., Boston, MA, USA).

The intensity of the metallic needles allowed determination of their exact final locations by inspection of the axial and sagittal planes at each insertion level (Figure 3A,C). The images were segmented in the three orthogonal planes (axial, sagittal, coronal). Semiautomatic threshold segmentation discriminated the “whitest” voxels (Figure 3 and Figure 4), creating the volumes of interest for vertebral structures and needles (Figure 3B,D,E and Figure 4B), as described previously [8]. After 3D reconstruction of the vertebral shape and needle locations, definitive insertion angles were measured in the axial and sagittal planes.

In thin patients, the skin is depressed around the spinous process (Figure 1B), reducing the (st) distance. A (st) correction (stc) was then added between the (st) measurement and the highest skin level, determined by the spinous process level, to obtain a parallel corrected distance (pcd)*,* which was comparable to (d) (Figure 1B). Finally, the distances (d), (st), and (pcd) were measured. The Pearson correlation index was calculated between those distances.

### 2.6. Anatomical Illustration of Needle Path and Affected Structures

Anatomical visualizations of structures related to the path traversed by the needle were prepared from the anesthesiologist’s point of view by a previously developed 3D PDF model [7,8], using the tools to make partially transparent the ligaments, bones and dural sac, for illustrative purposes.

## 3. Results

Success and redirection rates are detailed in Table 1. Successful punctures are illustrated in Figure 3 and Figure 4.

In cases with pre-estimation of (d), median needle punctures intended to be inserted perpendicular to the skin (78%) reached the spinal canal in most cases (93%). For 1 cm paramedian approaches inserted perpendicular to the skin (68%), success was noted in 94% of attempts, with final angles of 83.2°–95.9° to the axial plane. Needle redirection was required in 22% and 32% of attempts for median and 1 cm paramedian approaches, respectively; the corresponding final sagittal angles were up to 23.0° medially or 20.7° paramedially, with success rates of 75% and 88%, respectively (Table 1).

We achieved 100% success with paramedian approaches using individualized paramedian distances (0.6–2.0 cm) and sagittal angles (0.0°–15.2°). The best interlaminar window was identified at 2 cm paramedially (Figure 3C,D and Figure 4D).

Some needles became angled when being inserted in both the median and paramedian approaches, but in the observed cases tissue resistance directed the needle toward the dural sac (Figure 4C).

**Figure 5:**
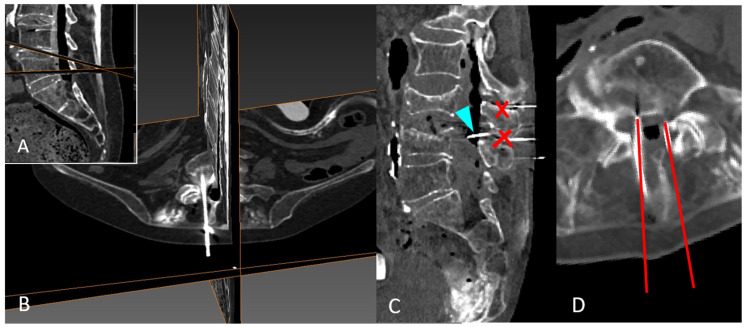
Representative images of unsuccessful needle insertions. (**A**) Sagittal view with axial and oblique planes in orange. The oblique plane follows the needle path that is shown in (**B**). (**B**): Oblique plane, following the needle path and oblique view of the sagittal plane, reproducing the anesthesiologist’s point of view: Vertebral body penetration occurred in this rotated spine after a straight and blind median needle insertion with a conventional cephalad angle. (**C**) Sagittal view of a spine with an osteoporotic compression fracture that reduced the interspinous space. If the axial plane below the upper spinous process was followed, the needle would contact the lower process, impeding the median approach (red crosses). The spinal canal could, however, be reached by a paramedian approach (arrowhead). (**D**) Needle locations inside the spinal canal, adjacent to the dural sac. The projection of the needle path is illustrated by red lines superimposed in the image.

Unsuccessful punctures had different causes. Blind cases coincided with cases presenting spine pathologies. One blind case presented a rotated spine in which the needle penetrated the vertebral body (Figure 5A). The other blind case had osteoporotic compression fractures that impeded the median approach at 0° due to contact with the adjacent spinous process; however, the paramedian approach allowed the needle to reach the spinal canal (Figure 5B). No median perpendicular needle insertions were successful in blind cases, and success was only achieved in 66% of cases after redirection. In 13.6% of median and 10% of paramedian punctures, the needle was directed straight forward and reached the spinal canal but not the dural sac (Figure 5C).

**Figure 6:**
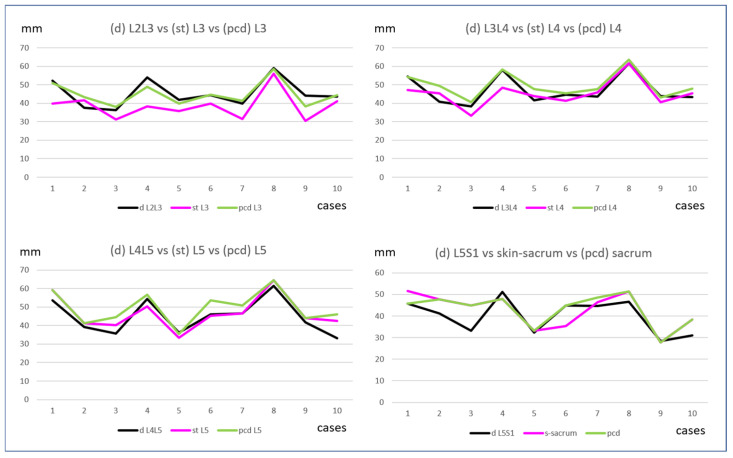
Comparison of the skin-to-dural sac distance (d), skin-to-transverse distance (st), and parallel corrected distance (pcd) at different intervertebral levels. The distances are presented in millimeters for each level across the 10 cases to better illustrate the correlation between these three parameters.

The distance (d) showed correlations of 0.91, 0.93, and 0.89 with the (pcd) at intervertebral spaces L4–L5, L3–L4, and L2–L3, respectively, but only 0.71 at the L5–S1 intervertebral space. The correlation between (d) and (st), without correction for thin patients, was 0.92, 0.81 at intervertebral spaces L4–L5 and L3–L4, respectively, but only 0.69 at L2–L3 and 0.71 at L5–S1. Nevertheless, the maximal absolute difference between (d) and (st) was 15.6 mm at L2–L3, with an average difference of 3.4 ± 2.7 mm at L5, 4.0 ± 2.8 mm at L4, and 7.6 ± 4.8 mm at L3 (Figure 6)

**Figure 7:**
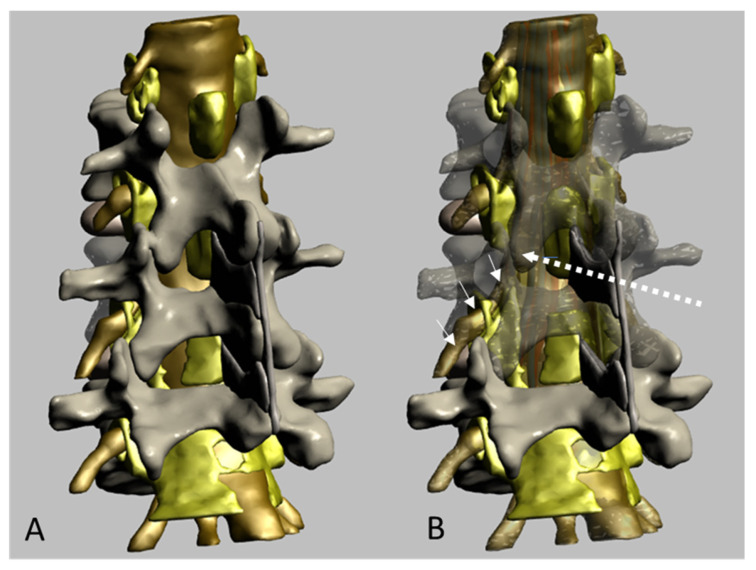
(**A**) Posterior oblique view of a freely available interactive 3D-PDF showing structures of interest for regional anesthesia. (**B**) Spinal lamina and dural sac have been made partially transparent to allow the visualization of cauda equina roots (motor roots illustrated in red, sensory roots in blue). The discontinuous arrow illustrates the needle path, reaching the lateral parts of the dural sac, where the roots with the corresponding nerve cuffs emerge through the intervertebral foramen (short white arrows).

The potential affectation of nerve cuffs and emerging nerve roots when needle remains lateral to the dural sac is illustrated in Figure 7.

## 4. Discussion

The novelty of the present study is that it showed a high success rate for needle insertion using a visual guide, with confirmation by CT scan, in flexed ex vivo samples that simulate clinical practice. This complements existing approaches to measuring angles by CT scan in standard decubitus positions [22,23]. Another novelty is the description of a high correlation between distances (d) and (st) -or (stc) in very thin patients-. The easier measurement of the (st) distance allows its use as a reference to calculate paramedian angles when (d) is not easily quantified due to calcification of the interspinous ligament, as is common in older people. [24]. Finally, the consistency of the viable perpendicular needle insertion, taking the upper process as reference, is another key finding of the study.

One limitation of this study is that the prone position of the flexed ex vivo lumbosacral trunk does not fully replicate the sitting or lateral decubitus positions typically used in clinical settings. However, we assumed that the spinal rearrangement and increased interspinous distance achieved by placing a support under the abdomen mimics the augmented interspinous distance observed in sitting patients flexed in a bent position. This assumption is crucial to demonstrate the consistency of viable perpendicular needle paths using the upper spinous process as a reference when vertebral bodies are preserved. Such a consistent perpendicular approach may facilitate median needle insertions without contacting the upper or lower lamina, addressing a common issue in blind needle insertions with a certain vertical angle that are still frequently intended nowadays.

It was also important to demonstrate that, once the skin-to-dural sac distance (d) is determined, the optimal paramedian angles would be symmetrical on both sides of a flexed ex vivo trunk. This symmetry allows the same trunk to be used for testing successful needle insertions at a 1 cm paramedian distance by following the inferred angle on one side, while also enabling individualized needle insertions at other paramedian distances on the opposite side. This approach optimizes the number of studied attempts per specimen.

Even if needle insertions are not performed at the same relative position that an anesthesiologist would insert them at, the aim was to demonstrate that inferred angles are only dependent on (d) and that successful insertions could be achieved by simple guidance, without specific training, since many successful 1 cm paramedian needle insertions were performed by the member of the team that was not an anesthesiologist.

Correct needle placement within the spinal canal after insertions at 0° in the axial plane is consistent with prior descriptions of successful attempts measured in the left lateral position [13], even in patients with scoliosis or spinal muscular atrophy, with 70% of success at the first attempt, when ultrasound is used to confirm the viability of the path [25]. However, the finding that osteoporotic compression fractures may lead to contact with the adjacent spinous process, thereby impeding the median approach, is also consistent with studies showing how spinous process deformations can leave no space for midline neuraxial punctures [23,26]. Furthermore, the reduced interlaminar space, which forces cephalad redirection, is typical in older people [17].

Needles being located in the spinal canal and not penetrating the dural sac may reflect the different consistency of ex vivo tissue in this experiment compared to clinical settings. However, anesthesiologists often encounter a scenario where they might be convinced of correct needle placement within the spinal canal, based on feeling a perforation of the flavum ligament, without cerebrospinal fluid leakage. If the needle tip is located laterally within the sac, it may also damage the nerve root at the emergence of the nerve cuffs, explaining clinical reports of radicular pain.

The occurrence of bone penetration in cases with severe osteoporosis suggests the potential for confounding in fragile patients. During blind approaches, for example, resistance may be interpreted as the flavum ligament or dural sac, but in fact, may correspond to penetration into deteriorated bone after traversing a thin flavum ligament. To prevent such scenarios, ultrasound may be used to identify the interspinous space before needle insertion or the skin to transverse process distance, allowing a more accurate estimation of skin-to-dural sac distances. The use of needles with marked distances could also help avoid needle advancement within deteriorated vertebral bodies after traversing the dural sac.

On the other hand, we previously noted that the conus medullaris may, in some cases, extend to the lower border of L2 [27]. Thus, inadvertent puncture of the conus medullaris could occur if the L3–L4 interspace is mistakenly identified as the L2–L3 or L1–L2 interspace, given the variability in the position of the intercrestal line, or Tuffier’s line, which may lead to misinterpretation of the selected spinal level during lumbar puncture [28]. Classical studies indicate that the anatomical interspace is correctly identified by palpation in only 29% of cases and is one level higher than assumed in 51% of cases, with discrepancies of up to two levels in some cases when compared with MRI findings [29]. Similar accuracy for palpation-based estimation (around one-third) has been reported in comparisons with ultrasound and X-ray imaging. Early studies showed that ultrasound improves accuracy in identifying intervertebral spaces by up to 70% [30], while recent studies still report that landmark-guided evaluation underestimates the intervertebral level in 30% of cases [4].

If the median approach is not viable in fractured or rotated spines, an individualized paramedian approach is appropriate. A recent meta-analysis suggested that the paramedian approach reduced the incidence of post-spinal headache and paresthesia [31]. However, paramedian approaches can be impaired by needle angulation within tissues, as reported in clinical practice [22,32]. Future guidance devices should improve the step between best-angle estimation by ultrasound and final needle introduction at the predetermined angle when the transducer is removed. Guiding needle introduction should also prevent needle angulation. At present, ultrasound is known to improve success during punctures [1,4,5,6]; it may help not only estimate the skin-to-dural sac distance but also the best angle for needle insertion and the correct intervertebral level.

The fact that final angles, intended to be perpendicular, showed a range of 83–96° is suggestive of the need to optimize needle insertions with guidance devices that minimize such variability, diminishing errors in needle insertion. Specific devices should also be designed to easily maintain the visualization of the optimal angle determined by the ultrasound probe, in cases of individualization of the paramedian approach, since a conventional protractor cannot be used in the sterile environment of the clinical settings. Such guidance devices could be also tested in flexed ex vivo trunks, with later CT assessment of needle location. Finally, such an approach could be considered in training courses, although a good coordination with CT facilities should be considered in that case. Future designs could take into consideration the possibility of needle insertions in lateral decubitus or in vertical positions simulating a sitting position.

Three-dimensional images have been shown to increase the understanding of the structures involved in spinal anesthesia [18,19,21]. Thus, the transparency of ligaments and vertebra has been decreased in our freely available model to illustrate the possible needle path and the potential to affect the cauda equina nerve roots and nerve cuffs. The 3D-PDF interactive model may be downloaded from the Public Repository of the University of Barcelona and runs in desktop computers with Acrobat Reader, allowing zoom, rotation, and clipping of the model, among other functions.

In conclusion, ultrasound can determine if the median approach is viable and if needles may be inserted at 0° relative to the axial plane, using the upper process for reference. If not, the paramedian approach offers an alternative, using visual guides according to the skin-to-dural sac distance or using the skin–transverse process distance as reference to calculate the paramedian angle. Approaching at an individualized paramedian distance may be necessary in rotated spines. Future efforts should consider device designs that facilitate the individualization of needle insertion, once the angle and depth of the needle path have been estimated, limiting needle penetration, and should seek to prevent needle angulation.

## Figures and Tables

**Table 1:** Needle insertion success rate by approach.

Needle Insertions	Median		Paramedian				
(Final Angles)			1 cm		Other cm		
Blind	*n* = 4	success	*n* = 7	success	cm	*n* = 4	success
Perpendicular (83.2°–95.9°)		0%		50%	1.43–1.63		100%
Redirected (61°–80°)	(75%)	66%	(43%)	66%	1.35–1.58	(50%)	50%
Ultrasound measurement of skin-to-dural sac distance	*n* = 18		Visual guide		Best ultrasound view		
			*n* = 25		cm	*n* = 4	success
Perpendicular (83°–96°)		93%		94%	1.95–1.99		100%
Redirected (69°–101°)	(22%)	75%	(32%)	88%	2.03	(25%)	100%

The table shows the number of needle insertions (n) and, in parentheses, the proportion of redirected insertions for each given (n). The range of final angles relative to the vertical axis is detailed. The success rate represents the percentage of needles correctly placed within the spinal canal.

## Data Availability

The original contributions presented in this study are included in the article. Further inquiries can be directed to the corresponding author(s).
